# Study on the restorative benefits of four behavioural patterns of urban landscape forests under seasonal change

**DOI:** 10.1038/s41598-025-09526-6

**Published:** 2025-07-07

**Authors:** Linjia Wu, Sirui Song, Yunlong Pan, Junyang Liu, Xin Zeng, Zongyuan Lu, Qibing Chen

**Affiliations:** 1 College of Art, Sichuan Tourism University, Chengdu, 610100 China; 2 Present Address: College of Landscape Architecture, Sichuan Agricultural University, Chengdu, China

**Keywords:** Urban landscape forests (ULFs), Behavioural patterns, Recovery, Physical and mental feedback, Psychology, Environmental sciences, Health care

## Abstract

Urban landscape forests (ULFs) are important green spaces that promote human well-being by providing health benefits and leisure opportunities. Most studies have concentrated only on health promotion differences in terms of plant community characteristics and have ignored the influence of a user’s own activity type. This study explores the restorative effects of different behavioural modes in deciduous ULFs. We chose 4 common behavioural modes, and a grouping experiment was conducted on a ginkgo scenic forest in Shuangliu Central Park, Chengdu, China. A total of 128 subjects were randomly divided into four gender-balanced groups. Physiological and psychological responses were evaluated using blood pressure, heart rate, electroencephalogram (EEG) measurements, and the Profile of Mood States (POMS) scale. The results revealed that the changes in systolic blood pressure and heart rate in the GL group decreased significantly, and diastolic blood pressure decreased significantly. In the monitoring of EEG changes, the α wave and β wave activity in the GS group and GW group were significantly increased. A comparison of the ANCOVAs among the four groups revealed that the α wave activity of the GS group was significantly greater than that of the other three groups (*p* < 0.001), the β wave activity of the GS group was significantly lower than that of the GW group, and the T–A mood values of the four behaviour pattern groups were significantly lower according to the POMS. According to the overall statistics of the available indicators, the health benefits of walking in autumn landscape forests are greatest, followed by sitting, lying and talking. The results of this research can encourage urban planners to consider appropriate behavioural guidance when developing nature tours or immersive nature projects on the basis of differences in behaviour patterns to gain more scientific insights into activity types.

## Introduction

### Urban landscape forest restoration: an important parameter of the environment and response mechanism

**Fig. 1:**
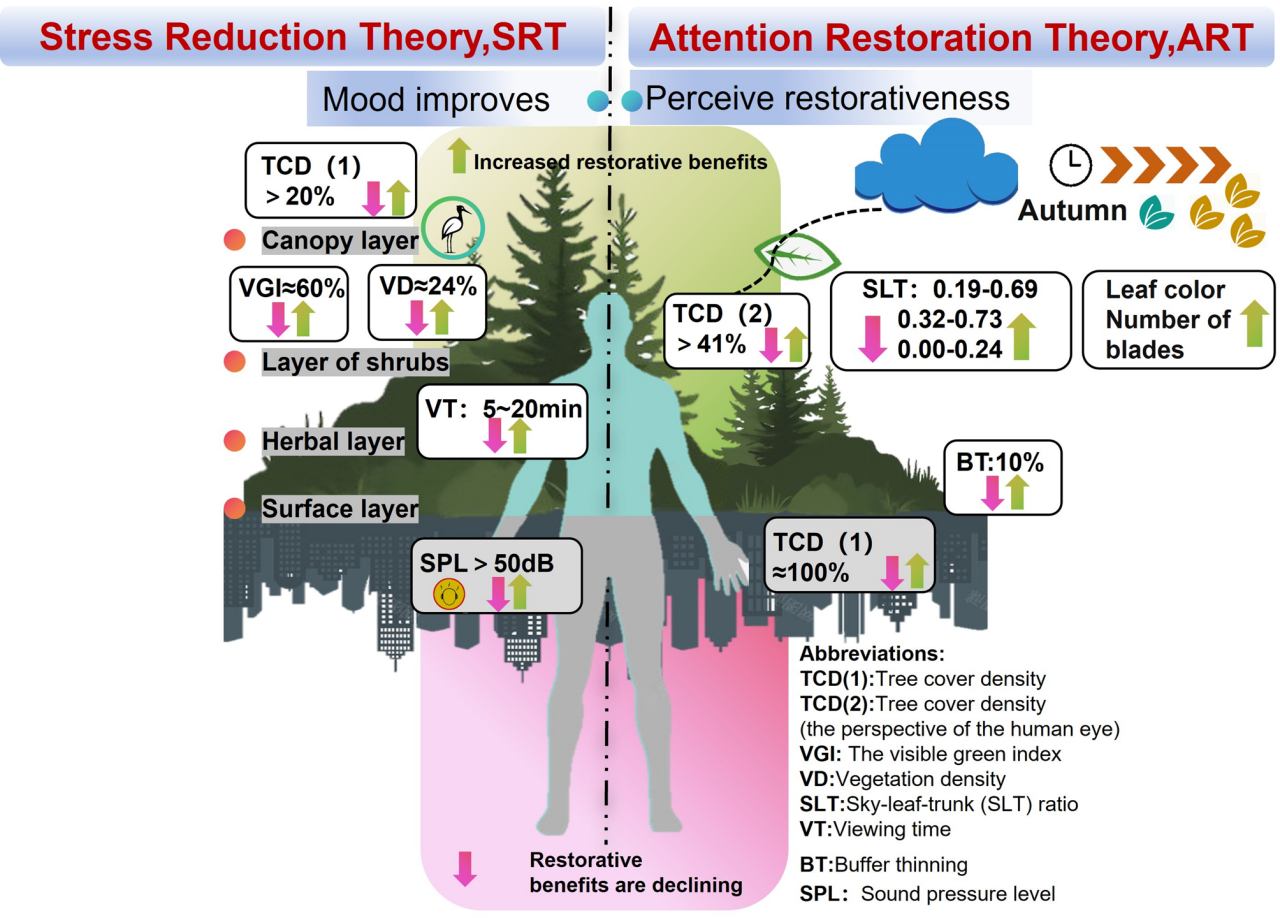
Benefits and parameters of ULFs restoration.

Cities around the world are promoting urban forests and establishing research organizations to improve the environment, economic conditions and the health of citizens^1^. For example, the International Union of Forest Research Organizations (IUFRO), the European Forest Research Institute, and the Urban Forest Research Center of the State Forestry Administration of China. It has been found that spending time in natural areas in urban settings can be used as a form of physical and mental recovery. Perceived pro-naturality in urban parks is a key mediator of vitality promotion^2^, and green exposure has a significant threshold effect on multidimensional human health^3,4^. Urban landscape forests (ULFs) are an important part of urban natural capital^5^. The basic elements of human well-being in urban planning and research include leisure environments. Natural areas in cities, such as ULFs, in the era of stock planning^6^ provide not only green spaces with rich ecological resources but also green space that urban residents have relatively easy access to. In terms of the health benefits of ULFs, people’s demand for forests in recent decades has shifted from traditional wood production to forest tourism and engaging in leisure in forests. It has been found that blood pressure, pulse, saliva production, and sympathetic nerve activity decrease significantly after visiting ULFs^7^ and that positive emotions^8,9^ increase; the findings regarding these health benefits, as well as other health benefits, provide solid physiological and psychological evidence for the benefits of ULFs. On the basis of the two major cornerstone theories of green recovery, attention recovery theory (ART) and stress reduction theory (SRT), the environmental attributes necessary for recovery include scope, escape, charm and compatibility^10^. Reducing stress is achieved through physiological (cardiovascular, skeletal muscle, and neuroendocrine system) and psychological (environmental cognition, fear, anger, and sadness) pathways after exposure to a natural space^11,12^. Therefore, existing theoretical research can be used to find effective common mechanisms and parameters associated with ULFs. These factors include lighting^13^, the proportion of element composition, conditioning^14,15^, availability^16^, the forest structure^17,18^, etc. (Fig. 1). The results of these studies revealed the extensive attributes of the high recovery power of ULFs: naturalization, biological diversity, colour enrichment, multiple layers, and some attributes that have dose effects^1^.

Although this overwhelming evidence indicates that the presence of natural spaces in cities is conducive to reducing pressure and restoring attention, the study period of almost all these studies was the spring and summer seasons, which are warm^19^, or periods that are conducive to plant growth, during which leaves are green. The dynamic changes in the season and the dynamic impact on user recovery have been ignored^20^. Studying the deciduous period may be important because it affects the length of the growing season, which affects all physiological processes such as photosynthesis and carbon dioxide exchange^21^. The changes in plants that occur during autumn have varying degrees of influence on human activities. Some studies have shown that the changes in leaf colour and quantity caused by seasonal differences in environmental conditions may lead to different potentials for recovery^20,22^. The colour of light green and green leaves in autumn can evoke the activation and relaxation of the brain^23^. Yellow leaves may indicate malnutrition in trees, which can lead to negative physiological reactions^24^. After taking a walk in a city park in autumn, people feel more comfortable, natural, relaxed, and energetic^25^. These studies mostly explored the relationship between the pressure level of a single season and its relationship with human perception. This research has focused on exploring the differential health benefits of urban scenic forests as plant communities with different characteristics under seasonal changes^26^. Few studies have focused on the value of different behaviours in health interventions. Many groups, including youth, are increasingly neglecting interactions with urban nature; this phenomenon is creating a generational amnesia and normalizing conditions that are detrimental to physical and mental health under urban stress^5^. Among public health prevention strategies, most studies have focused on promoting beneficial behaviours by intervening in socioeconomic and demographic factors^27^. Behaviour creates a bond between humans and nature. More suitable behaviour guidance with regard to ULFs is conducive to increased recovery. Therefore, more detailed behavioural model research should be regarded as an important resource for creating increasing amounts of urban green space.

### Behavioural mode and green recovery: an important but often overlooked aspect of exploring interrelationships

Natural recovery is a dynamic integration process, and the recovery benefits under different behavioural patterns may differ. Research supported by meta-analysis shows that behaviour is related to various urban environmental characteristics^28^, and these green environmental characteristics are the focus of current mainstream research. Basic research on self-development and health is important in urban green space construction^12^. Several studies have identified the link between conducting corresponding specific user group behavioural activities and recovery in urban green spaces. For example, in urban leisure green spaces, elderly people choose different types of activities on the basis of the complexity of the vicinity space and its degree of change^29^. There is a positive link between green space and walking behaviour in middle-aged people^28^, as green space is better at restoring attention and suppressing the autonomous nervous system than a city’s grey environment. Some studies have further explored two commonly used behavioural patterns in forests: the difference in recovery between watching and walking. Although similar recovery tendencies are shown in the same environment, the number of reactions is different^30^. It is believed that the viewing phase is almost a psychological experience and that the pedestrian stage includes psychological and physical interactions with the landscape. Walking can enhance vitality more than watching^31^, and viewing can provide more satisfaction^32^, including higher levels of “engagement” and “excitement” emotions^5^. Although the nonrandom design of these studies means that the results cannot be regarded as decisive evidence of causality, they provide evidence that different behavioural activities may mediate the impact of natural exposure on healthy well-being: these common behaviours are closely related to urban green space planning. Spontaneous human activities are a prerequisite for social activities^12^. The environment can inspire people to take action. Inducing desired behaviour is also conducive to those who enter green spaces to obtain positive results. Differences in group characteristics may affect perceived recovery results^3^, and only a few surveys have been conducted on the recovery benefits of different behavioural patterns in young groups in terms of walking and viewing. As an important period for individual social emotional development^33^, it is necessary to clarify the differences between broader behavioural patterns and green recovery to provide basic evidence for building a healthy urban environment.

The aim of this study is to explore the recovery benefits of different behaviours in deciduous ULFs. On the basis of the empirical use of ART and SRT in the research of green spaces in various cities, we believe that the natural elements of ULFs under seasonal changes facilitate recovery. The season has a significant effect in terms of counteracting the negative effects of the stress of urban life. The green environment featuring plants and the natural sounds of bird songs and wind^26^ have also been shown to have a special restorative effect. Therefore, in this study, these elements are incorporated into the selection of scenes. In terms of types of behaviour, according to the spontaneous and social activities affected by the environment in the city^34^, first, empirical evidence shows people choose walking and viewing activities, and second, some studies have proven the recovery efficacy of looking up at the sky, and lying in the forest and viewing the forest landscape also has health benefits^35^. The duration and methods of people’s interaction also have an impact on health^36^, and the “social” characteristics of the natural environment have been proven to be more liked by people^37^. Conversation is a key aspect of social interaction^34^. Therefore, we consider the common behaviours of sitting and walking, increased talking, and lying on the two behaviour modes of viewing and walking to observe the differences in recovery brought about by these different kinds of behaviour.

## Materials and methods

### Study sites

Chengdu (102°54 ~ 104°53 ′East, 30°05 ′ ~ 31°26 ′North) is located in southwestern China. It is the provincial capital of Sichuan Province. In April, the regional forest coverage rate in Chengdu increased to 59.5%, representing the typical level of forest greening in Chinese cities. Chengdu has a subtropical monsoon climate. The four seasons and the seasonal changes in plants are obvious. *Ginkgo biloba* are unique to China. The leaf colour in autumn is golden yellow, and the tree is present in Chengdu. It is planted in large quantities in the streets and parks of Chengdu, and it is a green tree species that the people of Chengdu are familiar with. Autumn is the golden period for appreciating ginkgo; the fact that this study includes autumn eliminates the influence of unfamiliar tree species and environments, which was present in other experiments.

**Fig. 2:**
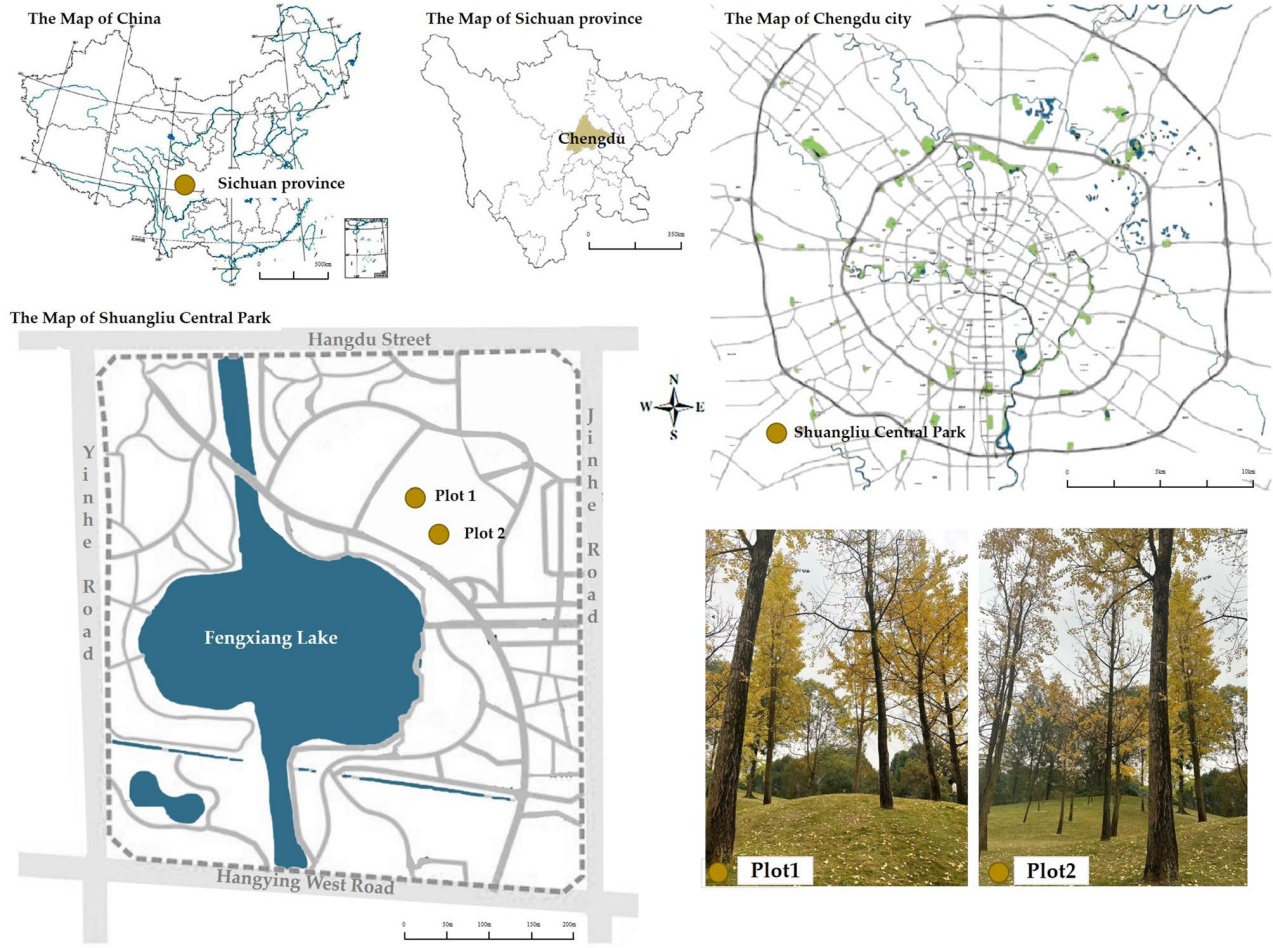
The location of the experiment(Map source: https://map.baidu.com/@11592686,3562668,13z).

Shuangliu Center Park is located in Shuangliu District, Chengdu. It covers a total area of 34 hectares. It is the central park of the eight major city parks in Shuangliu District and the largest park in or around Chengdu. There are not only rich natural resources in the park but also bodies of water and botanical gardens, in which more than 45% of the trees are ginkgo trees. Common urban green space elements such as pedestrians, trails, and signs can be seen in the site. For the purpose of the experiment, we chose an urban ginkgo forest with a relatively small area, few tourists, and a certain natural slope change as the study site. The site is in the green area near the east entrance of the park. The ginkgo green space is approximately 160 square meters long, with a total of 2 experimental points. The distance between the two experimental points is not greater than 15 m. The experimental time was November, and the ginkgo leaves were in their golden yellow season. The location of the experiment is shown in Fig. 2.

### Data collection

The assessment of recovery benefits in the field of environmental psychology is mainly based on ART and SRT^11,38^. Many feedback, response, and dose-based studies have been have conducted to understand the recovery process and role of the environment, which can provide a foundation for this study. The stimulus feedback path of urban green space recovery benefits is mainly through mental health, physical health, and social interaction effects^39^. Although previous research has focused on short-term tests, understanding the effect of social interaction requires a long-term stimulus feedback mechanism; therefore, the effect of social interaction is not considered in this study. The natural environment can induce side-in-see-out neurological activity, and its physiological changes reduce the sense of unpleasant pressure. Moreover, contact with the natural environment helps supplement targeted attention to a lesser degree^40^. Therefore, in this study, physiological and psychological indicators were combined to increase the completeness of the data collection.

In previous physiological measurements of restorative benefits, the physiological response to the five sensory-mediated stimuli were measured using brain activity (near-infrared spectroscopy [NIRS], electroencephalogram, etc.)^18^, autonomic activity (heart rate variability^12^, heart rate and blood pressure^35,41^, etc.), endocrine activity, and immune activity (natural killer cell activity, etc.)^42,43^. In this study, electroencephalography (EEG) data (α waves, β waves), heart rate, and blood pressure were used to assess physiological indicators of restorative benefits. EEG is a nonintrusive traditional brain cognitive process research method^12,18^. The ratio of different bands can also represent physiological feedback. β waves are usually related to alertness and awakening. α waves are usually related to relaxation. They are related to a state of pleasure, and θ waves are generated at a low level of physiological awakening. In this study, the Bitbrain can be monitored by a wearable brain electrical system with multiple mobile channels, and a total of 16 channels are monitored by the instrument. The electricity sampling frequency is 256–1000 Hz/per channel, and the resolution is 24 bits. Except for 16 EEG channels, the 16-bit inertia motion unit (IMU) (9-axis sensor) is used. Blood pressure, including systolic blood pressure and diastolic pressure, was measured with a blood pressure meter (OMRON, HEM-6322T, OMRON, Tokyo, Japan) with a blood pressure meter (BMP). When people are nervous, systolic blood pressure and diastolic blood pressure increase, and their pulse increases accordingly. Measuring blood pressure and pulse is a common method for determining the effectiveness of forest therapy^4^.

The abbreviated Profile of Mood States questionnaire (POMS) is another reliable and valid instrument for measuring mental state. It includes 40 adjectives rated on a 0–4 scale (0 = not at all, 4 = extremely), and these adjectives can be consolidated into seven effective dimensions, namely, Tension–Anxiety (T–A), Depression (D), Anger–Hostility (A–H), Vigour (V), Fatigue (F), Confusion (C) and Self-Esteem (S). POMS also includes three psychological indicators: positive, negative, and TMD (total mood distress index). TMD = 5 negative emotional scores and minus the sum of two positive emotions (energy, self-esteem) score + 100.

### Participants and experimental procedure

In laboratory experiments, most variables can be controlled or excluded, but onsite experiments in a natural environment can reflect genuine conditions rather than assumed conditions^44^. We use outdoor onsite experiments to collect data and evaluate recovery benefits. The age of participants leads to relevant biological differences in stress recovery^1^, so in this study, a typical youth group, namely, college students, is used as participants, and the age of all participants is between 18 and 25 years. The study lasted for one week, and all experimental processes were in line with the Herkinsin Declaration and received the approval of the Moral Ethics Association of the Academy of Art Design of the Sichuan Academy of Tourism. The experimental season was autumn, and the experiment was conducted from November 15 to November 20, 2023. The average temperature of the study area was 21 ± 2.0 °C, and the average humidity was 64.2 ± 3.1%. Given that confounding effects (such as noise) in large samples may influence the effects of interventions, many previous studies have used small sample sizes^6^. The maximum number of people who were tested per day was 25. We recruited 130 participants using a poster on campus. Before the experiment, the participants were surveyed for information such as their age, sex, and health status. All the subjects were right-handed. A total of 128 health college students participated in this study. The proportion of men to women was 1:1 (64 boys and 64 girls; average age: 22.53 ± 0.32).

Before the experiment started, the staff sent detailed instructions to the volunteers. The day before the experiment started, the volunteers were subjected to static exercise and allowed to drink and take medicine. The experiment is divided into four groups on the basis of different behavioural patterns: Group Sitting (GS), Group Walking (GW), Group Talking (GT), and Group Lying (GL). The participants are distributed randomly, though the number of men and women is the same in all groups, and work personnel supervise the experiment. There are two stages of the experiment, and volunteers are permitted to use smartphones during the experiment. The first stage was the preparation stage. The participants arrived at the east gate entrance square of Shuangliu Center. After reading the instructions, the participants signed the informed consent form and arrived at either of the two experimental points. The second stage is the experimental stage. After the participants arrived at the experimental location, they wear blood pressure monitors and EEG monitors, turn on the EEG records, and carry out distraction and pressure increase tasks on the side of the roadside, specifically 10 min of English dictation and numerical mental arithmetic. After the task was complete, the measurement of brain electrical activity was stopped, blood pressure and heart rate were measured three times, and a simple POMS questionnaire was completed. During this period, the physiological and psychological datasets collected from each group were used as covariance (baseline) data for statistical analysis.

**Fig. 3:**
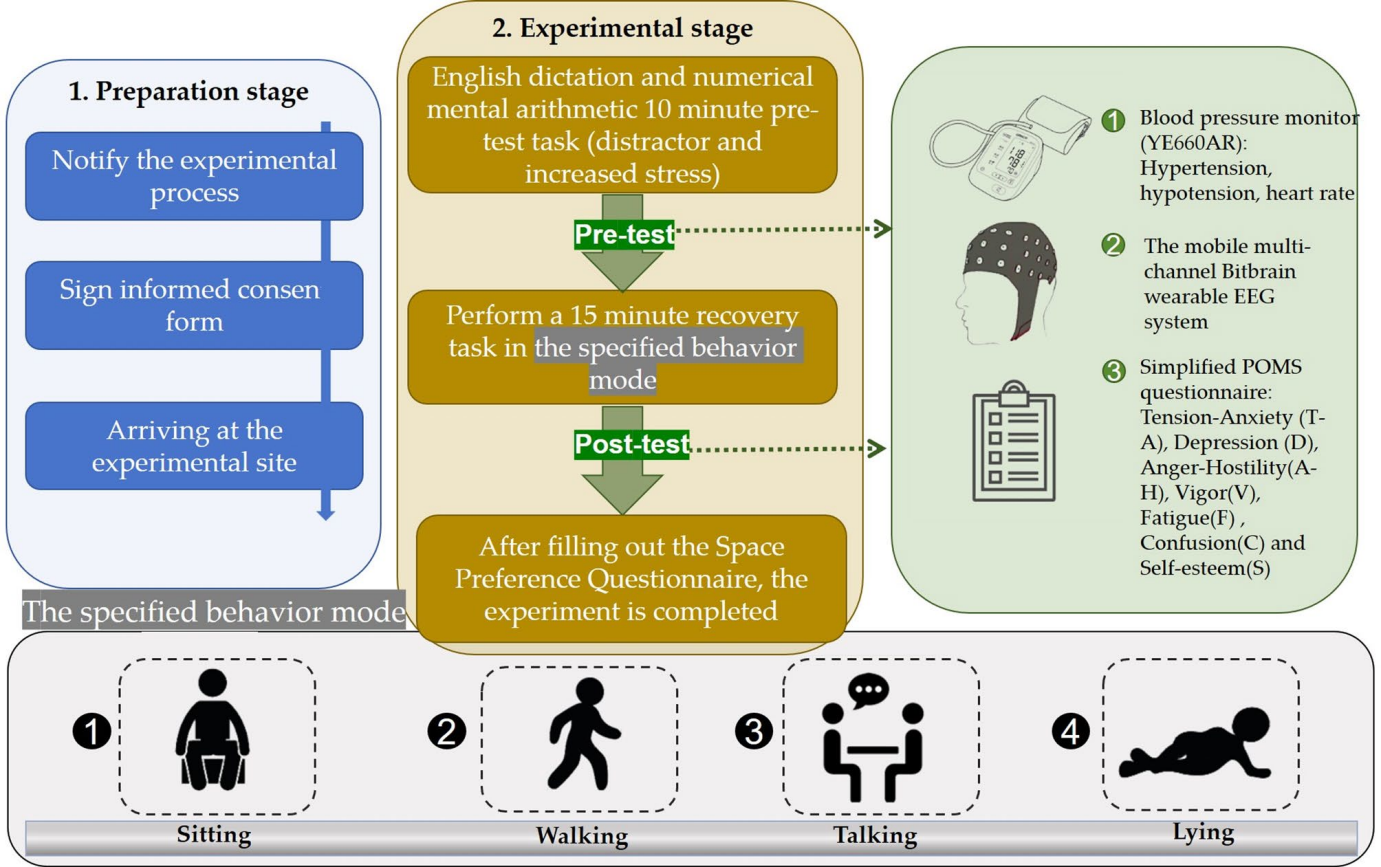
Schematic diagram of the experimental process.

Then, the staff and volunteers moved to the appropriate site to perform the second stage of the recovery task. According to previous research, the physiological effects of environmental stimuli occur after 4 min of exposure^45,46^, so the staff provided 15 min of an immersive experience. EEG data was recorded again with the EEG instrument. The conversation behaviour mode was facilitated by the staff, and the topic of the conversation was limited to landscapes. Recording brain and electrical data was stopped, and these measurement steps were repeated. Finally, the staff ended the experiment and paid the participants (Fig. 3). The research protocol and process were reviewed and approved by the ethics committee of the university and college (the ethics committee of the art college of Sichuan Tourism University), and the entire experimental process was in line with the norms and standards of the Declaration of Helsinki.

### Data analysis

Because participants were exposed to the same nature during the four behaviour modes, a validity test of the baseline (pretest data) and of the stimuli was performed before analysing the data. The paired sample t test was used to compare whether there was a significant difference in the data of each group and the pre- and posttest data. Since the sample size was less than 2000, the W test (Shapiro‒Wilk) was used before the paired samples to determine whether the difference followed a normal distribution, and if so, the paired samples t test was used to test for LDL levels. If not, a nonparametric rank sum test was used.

To exclude the confounding interference of uncontrollable factors, covariance analysis was used to analyse the differences in the effects of the physiological and psychological indicators of the four behaviours, and pretest data were used as covariates (baseline data) to eliminate the effects of slightly different baselines between different levels of individual stress and processing conditions. To satisfy the test hypothesis, the test statistics were calculated, and if *p* < 0.05, a postevent pairwise comparison was performed. SPSS 22.0 software was used for all analyses.

## Results

### Baseline check

The analysis of variance revealed that the average blood pressure (*p* = 0.17), heart rate (*p* = 0.24), α wave activity (*p* = 0.37), β wave activity (*p* = 0.21), and 8 mood statuses (*p* = 0.21) of the four experimental groups was 0.33. There was no significant difference between groups. After exposure to the source of stimuli, the average (*p* = 0.38), heart rate (*p* = 0.22), α wave activity (*p* = 0.21), β wave activity (*p* = 0.26), and 8 mood status statuses (*p* = 0.37) was not significantly different.

### Effects of different behaviour patterns on blood pressure and heart rate

**Fig. 4:**
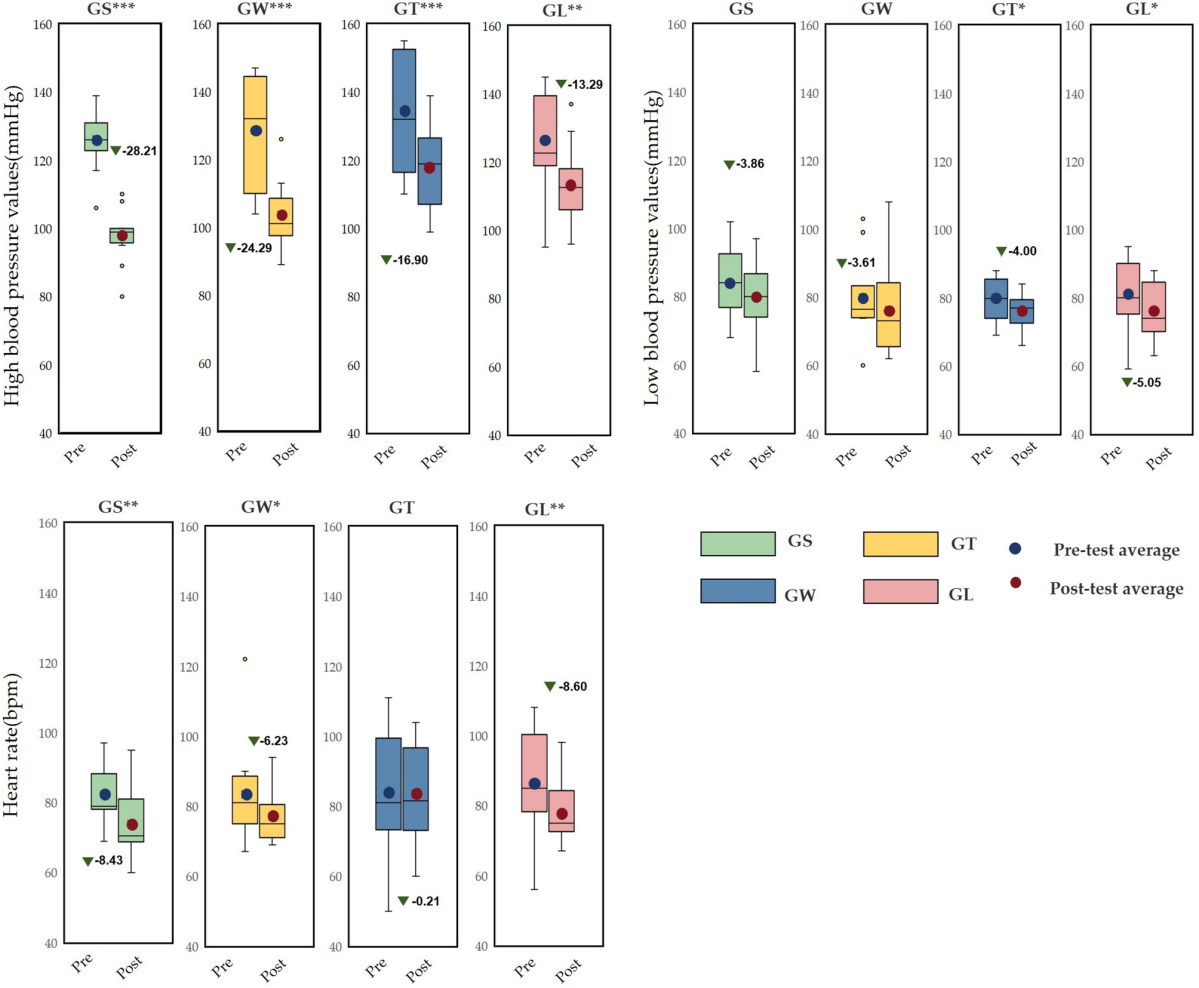
Pre- and post measurement effects of different behaviour patterns on blood pressure and heart rate (average ± standard deviation, *** *p* < 0.001, ** *p* < 0.01, *p < 0.05).

Figure 4 shows that in the GL group, blood pressure and heart rate changed significantly. The systolic blood pressure was 126.62 ± 14.66 mmHg before viewing, and after viewing, it was 113 ± 11.43 mmHg (*p* = 0.001**). The diastolic blood pressure was 81.61 ± 9.92 mmHg before viewing and 76.38 ± 8.02 mmHg after viewing (*p* = 0.013*). The heart rate was 86.53 ± 14.81 bpm before viewing and 78.15 ± 8.38 bpm after viewing (*p* = 0.007**). The systolic blood pressure in the GS group showed extremely significant changes before and after viewing, *p* = 0.000000358*** (before viewing: 126.07 ± 8.07 mmHg; after viewing: 97.85 ± 7.25 mmHg), the heart rate showed extremely significant changes before and after viewing, *p* = 0.001** (before viewing: 82.36 ± 7.95 mmHg; after viewing: 73.92 ± 9.98 mmHg), and there was no significant change in diastolic blood pressure, *p* = 0.057. The systolic blood pressure in the GW group showed extremely significant changes before and after, *p* = 0.00001*** (before viewing: 128.46 ± 16.75 mmHg, after viewing: 103.69 ± 9.24 mmHg), the heart rate showed significant changes, *p* = 0.032*(before viewing: 83.92 ± 13.98 mmHg, after viewing: 77.30 ± 7.33 mmHg), and there was no significant change in diastolic blood pressure (before viewing: 80.307 ± 12.31 mmHg, after viewing: 76.69 ± 14.19 mmHg). The systolic blood pressure in the GT group showed extremely significant changes; the systolic blood pressure was 134.46 ± 17.17 mmHg before viewing, 118.00 ± 11.96 mmHg (*p* = 0.000185***), and the diastolic blood pressure was 79.84 ± 6.59 mmHg before viewing, and 75.69 ± 5.48 mmHg (*p* = 0.045*) after viewing, and there was no significant change in heart rate (*p* = 0.736).

As shown in Table 1, ANCOVA of the three indicators revealed that there were no significant differences in hypertension, hypotension or heart rate among the four groups.

**Table 1:** Results of ANCOVA comparing blood pressure and heart rate parameters across the four behavioural modes (covariate, pretest, dependent variable: posttest).

Metric	Sum of squares	df	Mean square	F	Sig.	Partial η2
High blood pressure						
pretest	1939.60	1	887.35	30.47	0.053	0.054
behaviour	2535.502	3	53.23	13.27	0.065	0.242
error	3055.185	123	63.65			
R²=0.666 (adjR²=0.614)						
**Low blood pressure**						
pretest	2904.136	1	8.733	0.222	0.00001	0.606
behaviour	26.199	3	2904.136	73.737	0.881	0.014
error	1890.48	123	39.38			
R²=0.618 (adjR²=0.586)						
**Heart rate**						
pretest	2941.07	1	2941.70	63.20	0.0002	0.568
behaviour	568.35	3	189.45	4.07	0.12	0.203
error	2233.99	123	46.542			
R²=0.622 (adjR²=0.591)						

Note: * *p* < 0.05; ** *p* < 0.01; *** *p* < 0.001.

### The impact of different behavioural modes on brain waves

**Fig. 5:**
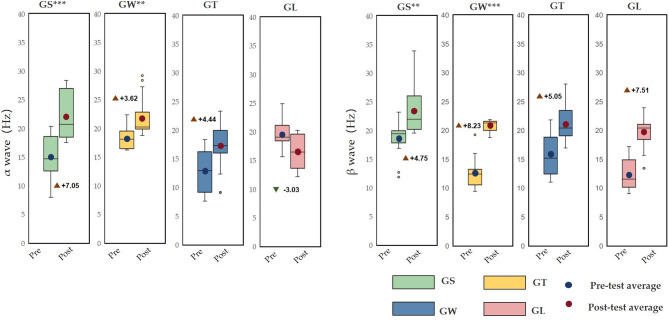
Effects of different behavioural modes on the pre- and posttests of α wave and β wave activity (average ± standard deviation, *** *p* < 0.001, ** *p* < 0.01, *p < 0.05).

**Fig. 6:**
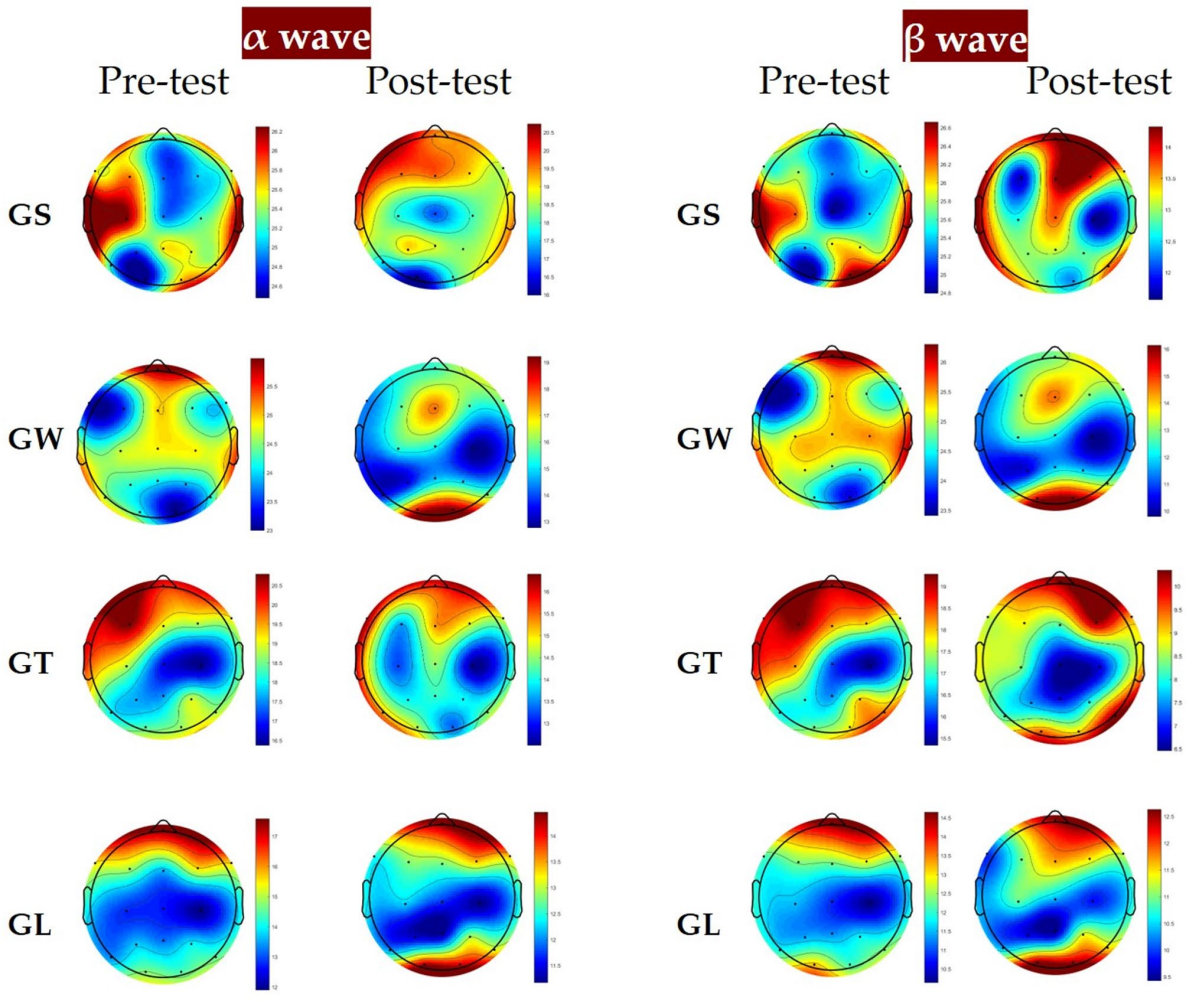
Distributions of α wave and β wave activity.

With respect to the α wave and β wave indicators, Fig. 5 shows that both those of the GS and GW groups increased significantly before and after watching. The α wave and β wave p values of the GS group and the GW group were both less than 0.001, and the α wave p value of the GW group and the β wave p value of the GS group were both less than 0.01. In the GS group, the α wave value was 14.96 ± 0.98 before viewing and 22.01 ± 1.10 after viewing, and the β wave value was 18.57 ± 0.81 before viewing and 23.33 ± 1.06 after viewing. In the GW group, the α wave value was 18.25 ± 0.49 before viewing and 21.97 ± 1.10 after viewing. The β wave value was 12.22 ± 0.80 before viewing and 20.80 ± 0.25 after viewing. The α wave and β wave values in the GT and GL groups decreased after viewing, but there were no significant differences, and the p values were greater than 0.05. Figure 6 shows that the power spectra of the four experimental groups of electronic data are divided into two frequency bands: alpha (8–12 Hz) and beta (12–30 Hz) and the power spectrum topographic diagram of the calculation average in each frequency band.

Table 2 shows that different behaviours have significant differences in alpha wave and β wave activity among the groups. For α wave activity, the groups were ranked GS > GL > GL > GT. For β wave activity, the groups were ranked GW > GL > GS > GT.

**Table 2:** ANCOVA was used to compare the results of the α wave and β wave parameters of the four behavioural patterns (symbol variables, pretest due to variables, posttest).

Metric	Sum of squares	df	Mean square	F	Sig.	Partial η2	Pairwise comparisons
**α waves**							
pretest	655.589	1	655.589	66.95	0.0000000001	0.582	GS> GW> GL> GT
behaviour	662.512	3	220.837	22.55	0.0000000003***	0.585	
error	479.024	123	9.792				
R²=0.695 (adjR²=0.669)							
**β waves**							
pretest	689.874	1	689.87	41.51	0.000000052	0.464	GW> G> GS> GT
behaviour	582.971	3	194.32	11.69	0.000007***	0.422	
error	797.64	123					
R²=0.533 (adjR²=0.494).							

**Fig. 7:**
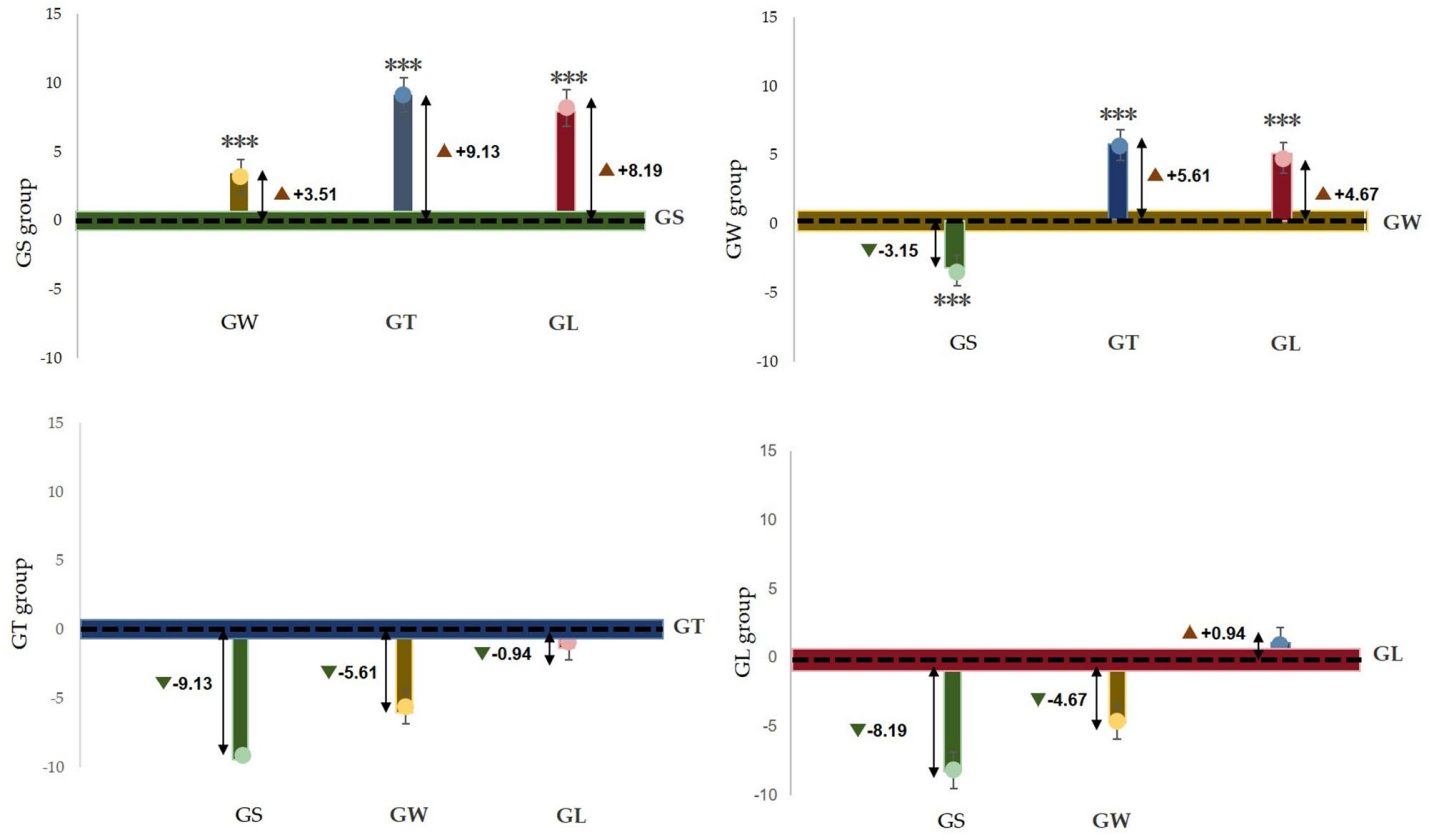
The average difference between the estimated marginal pairs of alpha waves.

**Fig. 8:**
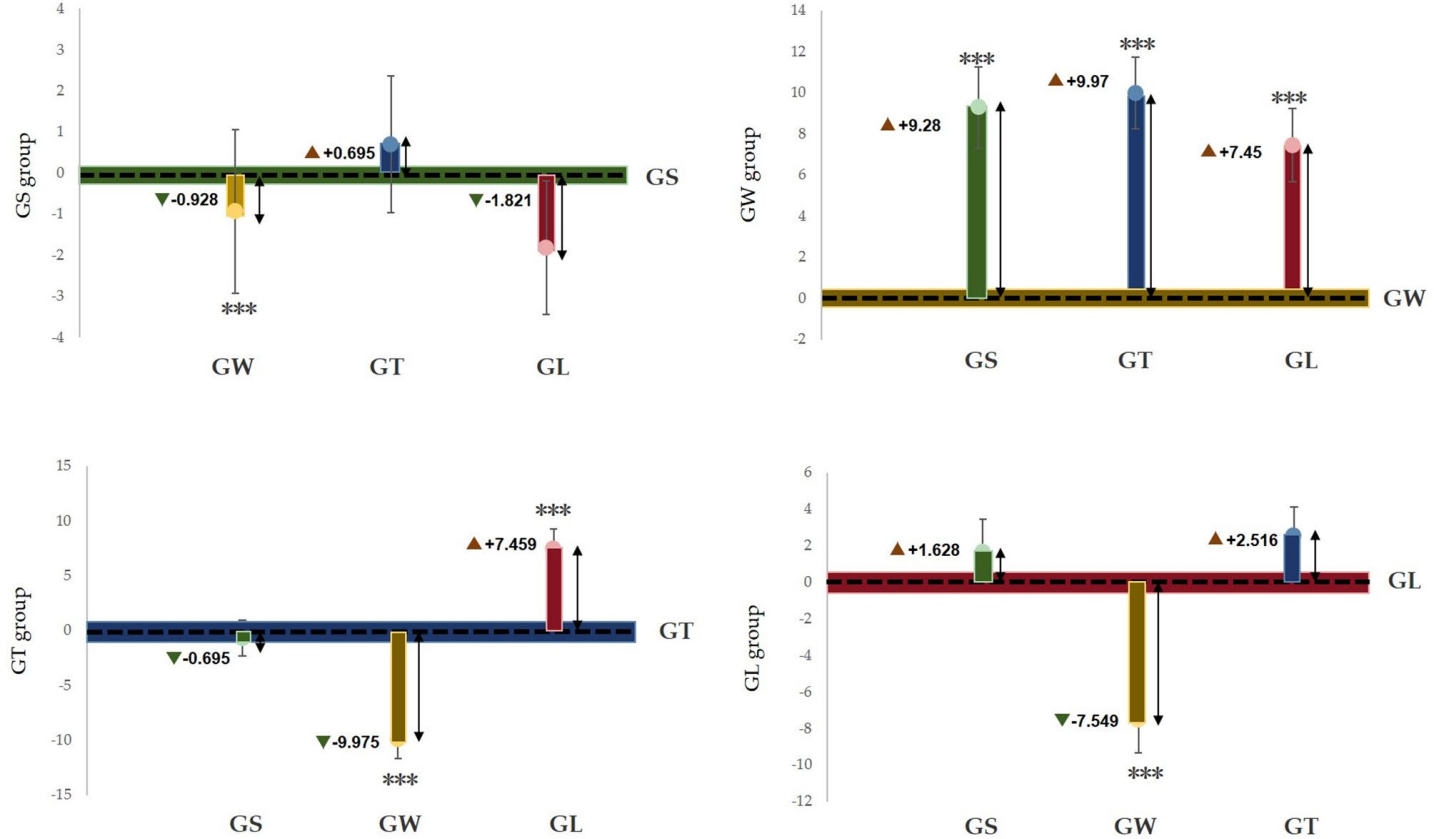
Based on the average difference between the estimated marginal pairs of β waves.

Figure 7 shows that the α wave index of the GS group was significantly greater than that of the other three groups (*p* < 0.001), and that of the GW group was significantly lower than that of the GS group, which was significantly greater than that of the GT group and GL group (*p* < 0.001). Figure 8 shows that the alpha wave activity of the GS group was significantly lower than that of the GW group (*p* < 0.001); the alpha wave activity of the GW group was significantly greater than that of the GS group, GL group and GT group (*p* < 0.001); and the alpha wave activity of the GT group was significantly lower than that of the GW group. Extremely higher than those in the GL group (*p* < 0.001). Compared with the GW group, the GL group had significantly lower values (*p* < 0.001).

### The influence of different behavioural patterns on psychology

**Fig. 9:**
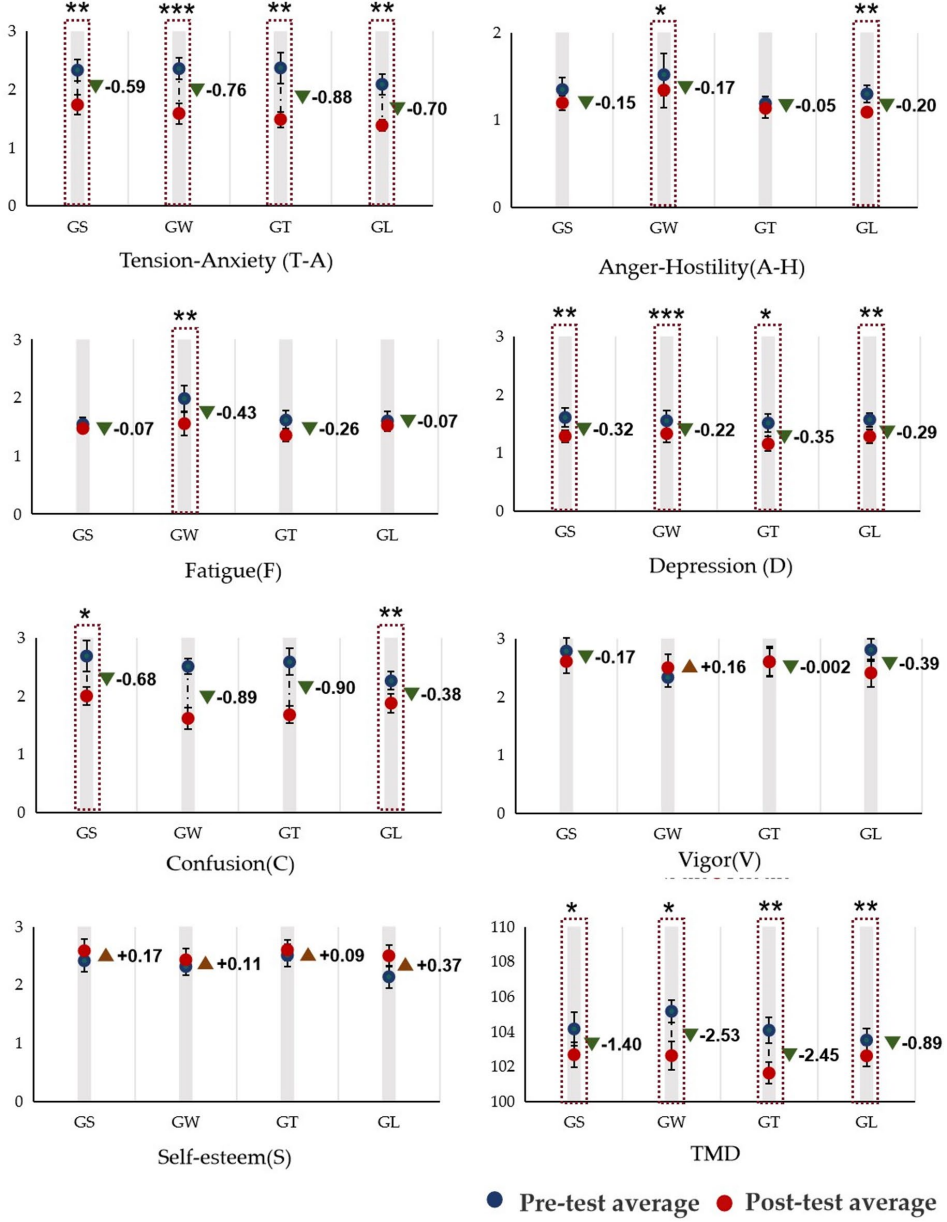
Effects of different behavioural patterns on the pre- and posttests of emotions (average ± standard deviation, ***p < 0.001, **p < 0.01, *p < 0.05).

Figure 9 shows 8 emotional values calculated by POMS. The T–A emotions decreased significantly in the four behavioural mode groups; the values in the GS, GT, and GL groups decreased by 0.59, 0.88, and 0.70, respectively, and the decline was significant (*p* < 0.01). The value in the GW group was 2.35 ± 0.18 before viewing and 1.59 ± 0.17 after viewing, and the T–A emotions decreased extremely significantly (*p* < 0.001). The A–H emotion of the GW group decreased significantly (*p* < 0.05), with a decrease of 0.17, and that of the GL group also decreased significantly (*p* < 0.01), with a decrease of 0.20. The F emotion decreased significantly (*p* < 0.01). The D emotion of all four groups declined significantly; the final values for the GS, GW, GT, and GL groups are 0.32, 0.22, 0.35, and 0.29, respectively. The C emotion of the GS group decreased significantly by 0.68 (*p* < 0.05), that of the GL group decreased significantly by 0.38 (*p* < 0.01), and there were no significant changes in the other two groups. There were no significant changes in the V emotion and S emotion. The TMD emotion of the GS and GW groups decreased significantly (*p* < 0.05) to 1.4 and 2.53, respectively; the GT and GL groups decreased significantly (*p* < 0.01) to 2.45 and 0.89, respectively.

**Fig. 10:**
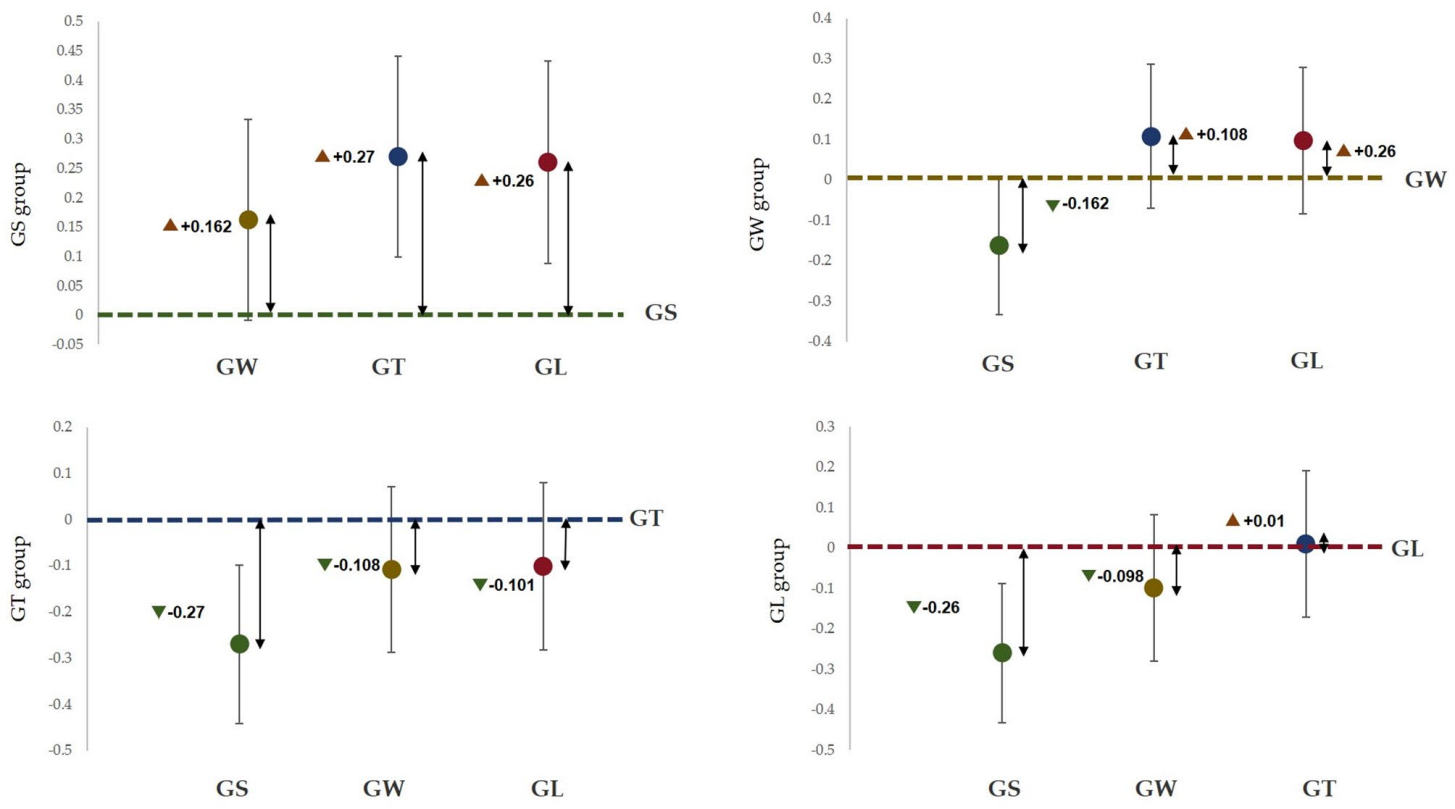
Comparison of the mean difference in T–A (tension–anxiety) sentiment between pairs based on the estimation margin.

**Fig. 11:**
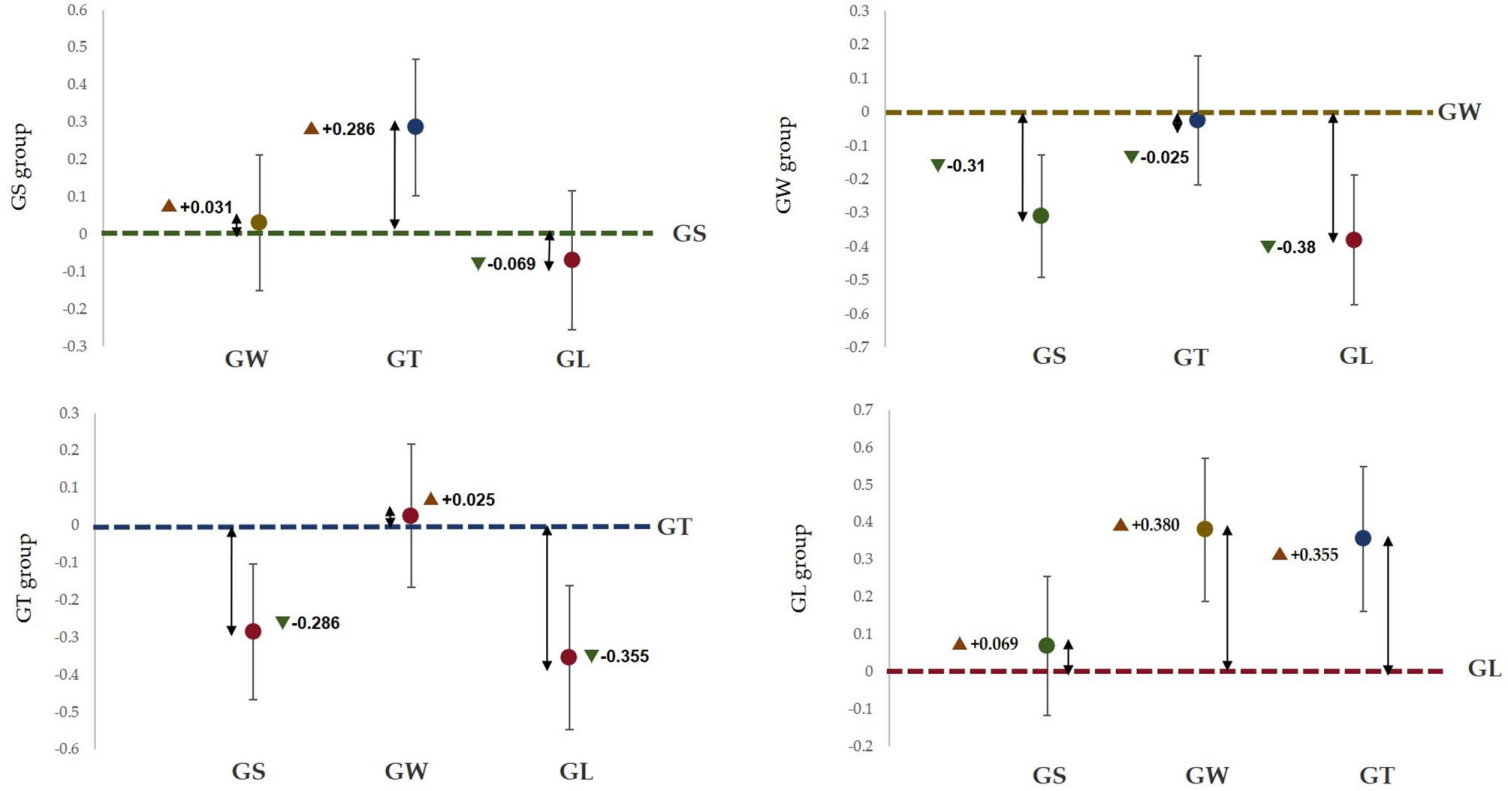
Comparison of the mean difference of the C (Confusion) sentiment on the basis of pairwise comparisons of the estimation margins.

Table 3 shows that there were significant differences in T–A and C emotions among the different behaviour patterns. For the T–A emotion, the groups ranked GS > GW > GT > GL, and for the C emotion, the groups ranked GL > GS > GT > GW. Figures 10 and 11 show the pairwise comparison mean difference and standard error, respectively, on the basis of the estimation margin.

**Table 3:** Results of the emotional parameters of the four behavioural modes of ANCOVA (Symbol variable, pretest. Due to variables, posttest).

Metric	Sum of squares	df	Mean square	F	Sig	Partial η2
**T–A (Tension–anxiety)**						
pretest	4.396	1	4.396	21.018	0.000031	0.296
behaviour	0.695	3	0.232	1.107	0.035*	0.062
error	9.736	128	0.191			
R²=0.338 (adjR²=0.285)						
**A–H(Anger–hostility)**						
pretest	7.653	1	7.653	183.656	0.00001	0.786
behaviour	0.108	3	0.036	0.863	0.466	0.049
error	2.083	128	0.042			
R²=0.7968 (adjR²=0780)						
**F(Fatigue)**						
pretest	5.411	1	5.411	23.129	0.000014	0.316
behaviour	0.324	3	0.108	0.462	0.71	0.027
error	11.697	128	0.234			
R²=0.328 (adjR²=0.275)						
**D(Depression)**						
pretest	6.501	1	6.501	80.086	0.00034	0.616
behaviour	0.161	3	0.053	0.662	0.58	0.038
error	4.059	128	0.081			
R²=0.624 (adjR²=0.594)						
**C(Confusion)**						
pretest	6.62	1	6.62	26.384	0.000005	0.345
behaviour	1.515	3	0.505	2.129	0.048*	0.113
error	11.863	128	0.237			
R²=0.391 (adjR²=0.342)						
**V(Vigour)**						
pretest	11.197	1	11.197	25.656	0.000006	0.339
behaviour	1.278	3	0.426	0.976	0.412	0.055
error	21.822	128	0.436			
R²=0.351 (adjR²=0.299)						
**S(Self-esteem)**						
pretest	6.637	1	6.637	20.749	0.000034	0.293
behaviour	0.776	3	0.259	0.498	0.046	
error	15.993	128	0.32			
R²=0.324 (adjR²=0.270)						
**TMD(Total Mood Distress index)**						
pretest	142.114	1	142.114	40.311	0.000013	0.446
behaviour	14.292	3	4.765	1.352	0.268	0.075
error	176.272	128	3.525			
R²=0.464 (adjR²=0.421)						

## Discussion

### Discussion of the differences in the physiological indicators of different behavioural patterns

**Fig. 12:**
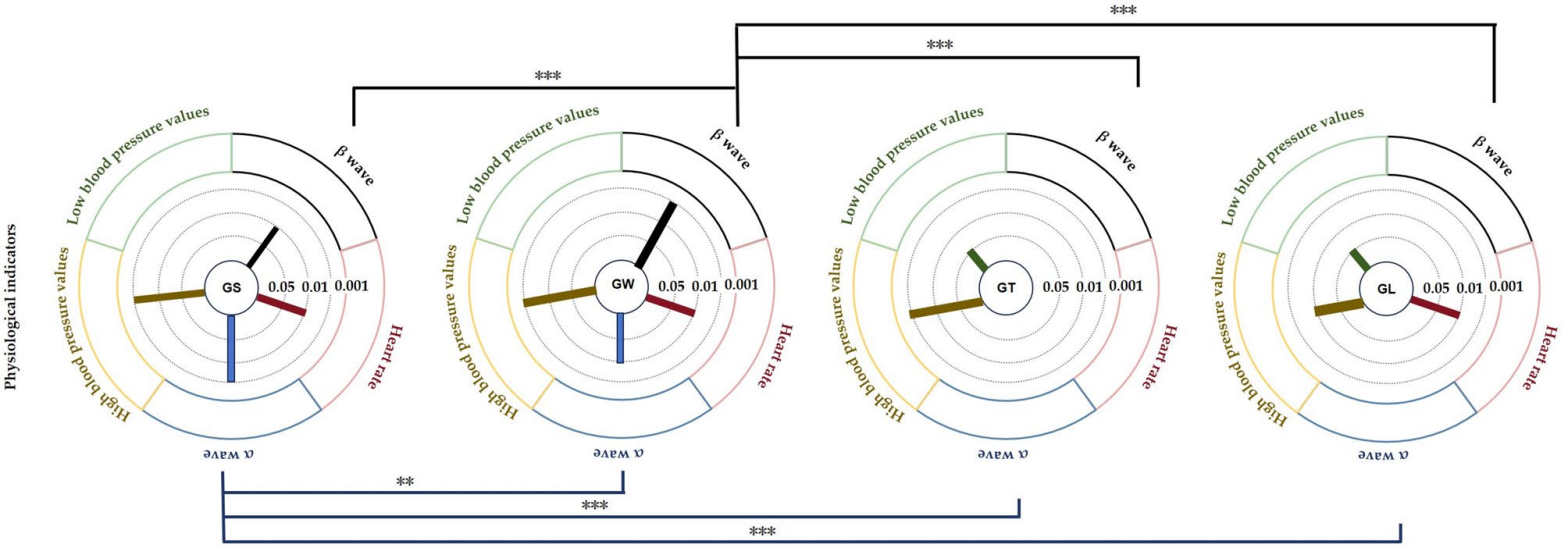
The physiological indicators of the 4 behavioural modes were measured before and after the experiment.

According to the seasonal natural exposure of different behaviours presented in Fig. 12, the four physiological indicators of sitting and walking behaviours are very significant. Because specific skills and equipment are not needed for sitting and walking, these behaviours are the most common personal behaviours in green spaces. Some studies have reported similar tendencies in terms of recovery. The accessibility of green environments makes it easier to walk, facilitating recovery^28^. Walking facilitates recovery more than viewing^31^. This finding is consistent with the significant increase in the α wave activity caused by sitting behaviour in this study (*p* < 0.001) and the significant increase in the β wave activity caused by walking in this study (*p* < 0.001). Blood pressure and heart rate are commonly used pressure indicators^35^. The results revealed that the systolic blood pressure during sitting and walking significantly decreased (*p* < 0.001), and the heart rate significantly decreased (*p* < 0.05). Blood pressure and heart rate are affected by stressful or intense experiences^42^, and season affects nature-mediated recovery^5^. Research has been conducted on the specific activities performed during a specific season in a natural space after the stress mechanism is triggered. This combination of nonattention environments is conducive to psychological and mental recovery^38^. Second, the essence of space perception is the process of obtaining information through spatial movement^35^. Environments that have a high-wavelength colour (such as yellow) can be more exciting than those of other colours, and this excitement can be quantified in brainwave data^47^. Compared with the other three behaviours, walking entails more physical exertion. Therefore, β wave activity is extremely significantly increased after walking. Additionally, changes in posture result in different visual perspectives. Visual information usually accounts for 70–80% of human perception^35^. Sitting and lying flat can be approximately understood as a psychological experience^30^. During quiet viewing, stress relief is consistent with psychological indicators.

We observed that lying behaviour was associated with significant changes in blood pressure and heart rate indicators, but there was no significant difference in EEG indicators. The ratio of trunk elements to single elements in the canopy landscape, which is part of the visual experience of being in green space, can affect EEG activity^35^. The single tree that was used in this study, i.e., ginkgo, did not cause significant changes in the EEG indices, which is consistent with the results of a previous study^35^. During conversation, only blood pressure showed a significant downwards trend, although some studies have shown that the it is possible for none of the studied indicators to decrease during conversation^48^. A possible explanation for this result is that talking to strangers (in experiments) may trigger people’s responses to unfamiliar stimuli, which can make people feel uncertain, though the impact on blood pressure is not significant. Although the desire for social interaction is one of the main factors driving demand for green space^49^, the potential perceived effects are not obvious. Figure 12 shows that pairwise comparisons reveal a significant difference in the four behavioural patterns. According to Kaplan ART theory^50^, attention and interest in the behavioural process of viewing and walking focus on the natural elements in the space (“charm”), leaf colour and green vegetation create a familiar and comfortable environment (“range” and “compatible”), and the two activities facilitate a temporary relief from the stress of urban life (“stay away”).

### Discussion of the differences in the psychological indicators of different behavioural patterns

**Fig. 13:**
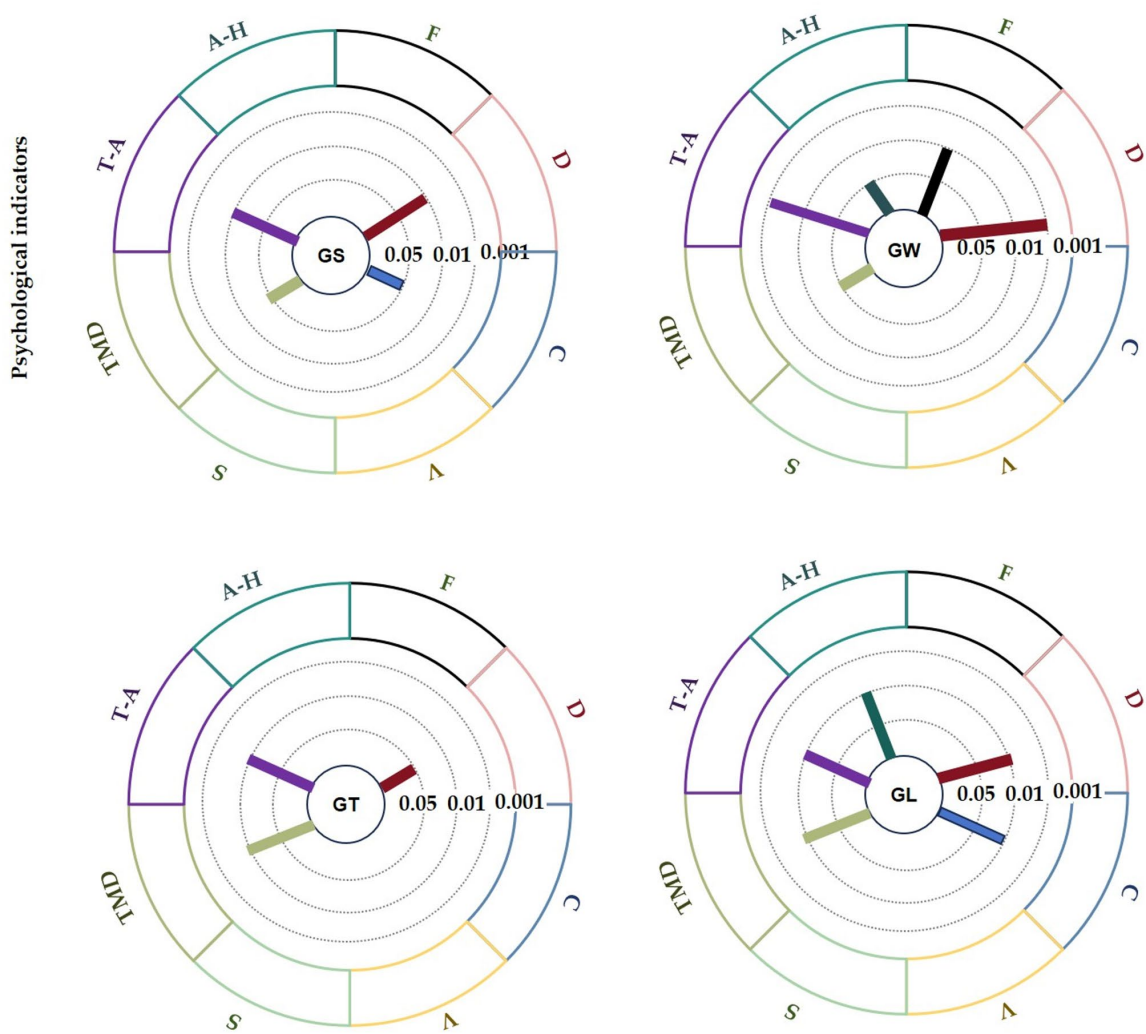
The psychological indicators of the 4 behavioural modes were measured before and after the experiment.

The four behavioural models significantly decrease in different degrees of indicators of negative emotions. This shows that the psychological response to being in nature in the context of this study is positive (Fig. 13). The core variable of this result may be that the behaviour mode is different, but there is no significant difference between the groups in the pair of pairs. The emotions of T–A, D, and TMD were significantly reduced (*p* < 0.05), extremely significantly (*p* < 0.01), and extremely significantly (*p* < 0.001) as a result of the four behaviours, which was consistent with the significant decreases in blood pressure and heart rate indicators shown in Fig. 12. In psychological cognition, participants focus on unique or interesting landscape elements in their surroundings^51^, and the key structural factors of psychological stress in pure forest space are “spatial diversity” and “closure degree”^52^. The plant species, landscape content and colour of the space in which the participants engaged in the four behaviours were the same, which may not be sufficient to overcome the intergroup differences in the benefits of inducing psychological relaxation. The three interrelated dimensions of the psychological response to exposure to nature, namely, obsession, coherence and humanity, build on the psychological responses to visual information and a warm natural environment, which produces similar positive psychological benefits when engaging in various behaviours^9^.

According to the SRT and ART theories, under the premise that the landscape space is easy to understand and link, if landscape content is easy to understand or relatively simple, it will also be judged as positive (or interesting) psychologically in the observation process^21^, which is consistent with this result. That is, the four behavioural patterns all show different degrees of positive emotions. Interestingly, we did not find an increase in vitality (V) or self-esteem (S) as a result of positive emotions. During the experiment, we used pretest experiments with increased attention and pressure. Certain psychological cognitive mechanisms were active in the observation process. It is not enough to trigger the enhancement of vitality or dignity. Beautiful scenery has a strong impact on human health and vitality^53^, and visiting urban forests can promote and enhance vitality^54^; however, this is a duration test in which feedback may not be suitable for this short-term stimulus. Some studies have also shown that after individuals walk in urban parks in autumn, they feel more energetic^7^. This study reveals a decrease (though not a significant one) in the calculation of psychological indicators, which shows that the human response to nature is psychologically and emotionally elastic^51^ and that the emotional feedback of vitality and dignity may not change significantly due to the characteristics of the behaviour that people engage in and differences in short-term exposure to stimulus.

### Behavioural pattern guidance of deciduous ULFs based on research results

Environmental and landscape factors can change behaviour in a person without them intending to^55^, and behaviour is an important interaction mode between humans and nature. Dynamic stimuli prompt more natural viewing behaviours than static stimuli do^13^, and walking in this study produced the best feedback in terms of physical and mental indicators, followed by sitting, lying, and talking. Since human automaticity is likely to be an adaptive function^56^, according to the results of this study, it is recommended that, in addition to providing the necessary rest facilities and keeping the park visually attractive, cleanliness and safety should be maintained, and guidance on walking in public natural spaces should be provided. The relatively positive impact of formal and informal trails on forest loss and tree structure has been confirmed^19^, and measures based on this conclusion include increasing the amount of paths and walking through grasslands to improve the human leisure of parks^57^. Visitors should be encouraged to increase the amount that they walk, while also viewing nature, by providing a sequence of scenic locations. Smart management should be used to encourage users to participate in the national fitness strategy. Second, satisfying and communicating intentions are important antecedents of behavioural decision-making^58^. Perceived value has a great positive effect on behavioural intention and satisfaction. Previous studies have shown that the value of stable nontimber forest products can often increase the perceived value of intact forests and motivate people to follow sustainability and conservation goals^59^. In combination with the goal of having healthy green spaces, park employees can educate guests about the sustainable products that can be produced in ULFs, and informational plaques can be added to increase the perceived value of the park to visitors. The results of this study also revealed more positive physiological and psychological feedback, and the facilities that allow visitors to comfortably lie down are popular, further validating the exact benefits of behavioural diversity for health. Our feedback results with regard to lying down are consistent with previous research, and lying down provides a new visual perspective. We recommend adding gently sloped hills with grass or slanted seats to encourage guest to lie down, thereby enabling visitors to obtain the health benefits of looking up at the canopy landscape, and setting up corresponding facilities at reasonable points at tree trunks^35^.

## Conclusion

This study, through empirical evidence provided by natural experiments, contributes to a more comprehensive understanding of the relationships between different behavioural patterns and restorative benefits under seasonal green exposure. The interdependent relationship between human and environmental factors^28^ can compensate for the promotion of the fair use of social green space. This study further confirms the positive relationship between human behaviour patterns and the environment, providing evidence that while the positive performance of various behaviours was consistent in field trials, walking achieved better physiological and psychological restorative effects than sitting. There were also significant differences between groups; for example, there were extremely significant differences in α wave activity caused by sitting behaviour compared with the other three behavioural patterns. Walking resulted in an increase in β wave activity. On the basis of the limited results of this study, we believe that short-term seasonally different behavioural patterns under stress stimulation may lead to significant feedback on physiological indicators (such as blood pressure, heart rate, and EEG activity), but the differences in psychological feedback mechanisms in the same type of green environment are not significant. In terms of different types of seasonal green environments, any of these behaviours may produce healthy results, and cities should create more green spaces. Further research into behavioural patterns would provide a “snapshot” of the health effects of exposure to green spaces, and on the basis of the research results, we advocate for increased accessibility of green spaces, the addition of trails, and healthy and intelligent interactive measures for new parks to create experiences and result in superior health benefits. Informational plaques that display the value of nontimber trees in ULFs should be added to increase the perceived value of ULFs.

## Limitations

The following shortcomings remained in this study. First, the study population was limited to young people, and although the biological differences of the population were reduced, the perceived and experienced behavioural effects between individuals were not discussed further. There are no data on social background and age breakdown in the study of the effects of different behavioural patterns on functional processes, and this unobserved confounding information may be ignored. Second, this study was limited to areas with significant seasonal colour variations, and although the study emphasized the suggestion of cue management for behavioural problems, it seems to be more applicable to the northern subtropical region. Further comparative studies are needed to determine whether the results of this study are applicable in tropical green areas.

## Data Availability

All data generated or analysed during this study are included in this published article [and its supplementary information files].
